# Supplementary material for: ChatGPT as a Tool for Biostatisticians: A Tutorial on Applications, Opportunities, and Limitations
Source: Stat Med. 2025 Oct 23;44(23-24):e70263. doi: 10.1002/sim.70263 (PMC12548020; doi:10.1002/sim.70263)
Supplement: Supplementary file 1 — Data S1. Supporting Information. [file SIM-44-0-s001.zip › Supplementary Materials Additional.zip!/3.7_Translation_Between_Multiple_Statistical_Programming_Languages/3.7.2_SPSS_to_R/3.7.2_Translation_Chat9.pdf]

R 9

Clear memory

I can't clear memory directly, but you can manage or clear your memory by following these steps:

1. Go to **Settings**.
2. Click on **Personalization**.
3. Select **Memory**.
4. From there, you can view what I remember and choose to delete specific items or clear everything.

Let me know if you'd like help navigating there!

Take the role of an expert biostatistician for this chat

Got it — I'm now in expert biostatistician mode. Whether you need help with study design, statistical modeling, clinical trial analysis, survival data, or interpreting biomedical data, I've got you covered. What are we diving into?

I am running the following analysis in SPSS:
DATASET ACTIVATE DataSet5. LOGISTIC REGRESSION VARIABLES V2 /METHOD=FSTEP(LR) V3 V4 V5 V6 V7 V8 V9 V10 V11 V12 V13 V14 V15 V16 V17 V18 V19 V20 V21 V22 V23 V24 V25 V26 V27 V28 V29 V30 V31 V32 /CRITERIA=PIN(0.05) POUT(0.10) ITERATE(20) CUT(0.5).
Translate this into R code.
The data is loaded into R with
temp <- tempfile()
download.file(
"https://archive.ics.uci.edu/static/public/17/breast+cancer+wisconsin+diagnostic.zip",
temp
)
data <- read.table(unz(temp, "wdbc.data"), sep = ",")
data$V2 <- data$V2 == "M"
unlink(temp)

Great — you're running a **stepwise logistic regression** using **forward selection (likelihood ratio)** in SPSS, with a dependent variable `V2` (converted to a binary outcome where `"M"` = TRUE) and predictors `V3` to `V32`. You're setting `PIN = 0.05` and `POUT = 0.10` as entry/removal thresholds for the stepwise selection.

Here's the equivalent R code using `step()` from base R, which performs stepwise model selection by AIC. While it's not exactly the same as SPSS's likelihood ratio test, it's the closest built-in method. We can also emulate likelihood ratio-based selection using `stepAIC()` from the **MASS** package (which is more similar in logic).

🔁 Equivalent R Code

```r
# Load required package
library(MASS)

# Set up the data: convert V2 to binary factor for logistic regression
data$V2 <- as.factor(data$V2)

# Build the null (intercept-only) model
null_model <- glm(V2 ~ 1, data = data, family = binomial)

# Build the full model with predictors V3 to V32
full_model <- glm(V2 ~ ., data = data[, 2:32], family = binomial)

# Run forward stepwise selection (similar to METHOD=FSTEP(LR) in SPSS)
stepwise_model <- stepAIC(null_model,
                          scope = list(lower = null_model, upper = full_model),
                          direction = "forward",
                          trace = TRUE)
```

Notes:

- `stepAIC()` uses AIC as the selection criterion, which often aligns closely with likelihood ratio testing.
- If you want stricter control with `PIN` and `POUT` p-value cutoffs, you'd need to write a custom stepwise function, since R's built-in methods do not let you specify p-value thresholds directly.
- The data range `data[, 2:32]` includes `V2` to `V32` — `V2` is the outcome, `V3:V32` are predictors.

Do you want help modifying this for stricter inclusion/removal based on p-values instead of AIC?

Yes

Perfect — to match SPSS's stepwise logistic regression with `PIN(0.05)` and `POUT(0.10)` based on **p-values of likelihood ratio tests**, we'll need to write a **custom forward stepwise logistic regression function** in R. This function will:

- Start with a null model (intercept only),
- At each step, test each remaining predictor using likelihood ratio test (`anova(..., test = "Chisq")`),
- Add the predictor with the lowest p-value if it's < 0.05 (`PIN`),
- After each addition, test whether any included variable has p > 0.10 (`POUT`) and remove if so,

- Repeat until no more predictors can be added or removed.

Here's a working version:

✅ Custom Forward Stepwise Logistic Regression by p-values

```r
stepwise_lr <- function(data, response, predictors, pin = 0.05, pout = 0.10) {
  included <- c()
  remaining <- predictors
  formula_base <- as.formula(paste(response, "~ 1"))
  current_model <- glm(formula_base, data = data, family = binomial)

  repeat {
    # Forward step: test all remaining predictors
    pvals <- sapply(remaining, function(var) {
      temp_model <- update(current_model, paste(". ~ . +", var))
      test <- anova(current_model, temp_model, test = "Chisq")
      test$`Pr(>Chi)`[2]  # p-value for likelihood ratio test
    })

    min_pval <- min(pvals)
    best_var <- remaining[which.min(pvals)]

    # If best variable meets PIN threshold, add it
    if (min_pval < pin) {
      included <- c(included, best_var)
      remaining <- setdiff(remaining, best_var)
      current_model <- update(current_model, paste(". ~ . +", best_var))
    } else {
      break  # no variable meets entry criterion
    }

    # Backward step: check if any included variable should be removed
    pvals_included <- sapply(included, function(var) {
      reduced_model <- update(current_model, paste(". ~ . -", var))
      test <- anova(reduced_model, current_model, test = "Chisq")
      test$`Pr(>Chi)`[2]  # p-value for LRT
    })

    max_pval <- max(pvals_included)
    worst_var <- included[which.max(pvals_included)]

    if (max_pval > pout) {
      included <- setdiff(included, worst_var)
      current_model <- update(current_model, paste(". ~ . -", worst_var))
    }
  }

  return(current_model)
}
```

📦 Use it on your dataset

```r
# Prepare data
data$V2 <- as.factor(data$V2)
predictors <- paste0("V", 3:32)

# Run the custom stepwise logistic regression
model_final <- stepwise_lr(data = data, response = "V2", predictors = predictors)
```

```
# Summary of final model
summary(model_final)
```

Let me know if you'd like to also add output like AUC, confusion matrix, or plot coefficients.
